# QTcNet: a deep learning model for direct heart rate corrected QT interval estimation

**DOI:** 10.1093/europace/euaf274

**Published:** 2025-10-27

**Authors:** Lucas Plagwitz, Florian Doldi, Jannes Magerfleisch, Maxim Zotov, Lucas Bickmann, Dominik Heider, Julian Varghese, Lars Eckardt, Antonius Büscher

**Affiliations:** Institute of Medical Informatics, University of Münster, Albert-Schweitzer-Campus 1/Building A11, Münster 48149, Germany; Clinic for Cardiology II: Electrophysiology, University Hospital Münster, Albert-Schweitzer-Campus 1/Building A1, Münster 48149, Germany; Clinic for Cardiology II: Electrophysiology, University Hospital Münster, Albert-Schweitzer-Campus 1/Building A1, Münster 48149, Germany; Institute of Medical Informatics, University of Münster, Albert-Schweitzer-Campus 1/Building A11, Münster 48149, Germany; Institute of Medical Informatics, University of Münster, Albert-Schweitzer-Campus 1/Building A11, Münster 48149, Germany; Institute of Medical Informatics, University of Münster, Albert-Schweitzer-Campus 1/Building A11, Münster 48149, Germany; Institute of Medical Data Science, Otto-von-Guericke University Magdeburg, Leipziger Str. 44/Building 2, Magdeburg 39120, Germany; Clinic for Cardiology II: Electrophysiology, University Hospital Münster, Albert-Schweitzer-Campus 1/Building A1, Münster 48149, Germany; Institute of Medical Informatics, University of Münster, Albert-Schweitzer-Campus 1/Building A11, Münster 48149, Germany; Clinic for Cardiology II: Electrophysiology, University Hospital Münster, Albert-Schweitzer-Campus 1/Building A1, Münster 48149, Germany

**Keywords:** 12-lead ECG, Deep learning, Artificial intelligence, Deep regression, QT interval estimation, QTc measurement

## Abstract

**Aims:**

Automated QTc measurements from commercial ECG systems often diverge from expert readings. We developed *QTcNet*, a deep learning model trained and validated on multiple large ECG datasets to improve automated QTc measurement accuracy.

**Methods and results:**

*QTcNet* employs a regression-based convolutional neural network architecture. It was trained on 120 300 algorithm-labelled ECGs (60 150 from an internal hospital cohort and 60 150 from the MIMIC-IV dataset) after correction for a vendor-specific +15 ms bias. Performance was evaluated against expert QTc measurements in three independent datasets: PTB Diagnostic ECG Database (*n* = 100 ECGs in validation set), QTcMS (*n* = 210), and ECGRDVQ (*n* = 5219). The effect of fine-tuning on cardiologist-annotated ECGs was tested in the PTB database (*n* = 449 in fine-tuning set). Model explainability analyses were performed with Integrated Gradient maps. *QTcNet* reduced cross-cohort mean absolute error (MAE) from 23.4 to 13.4 ms and root mean square error (RMSE) from 40.1 to 22.1 ms, almost halving large (>50 ms) outliers. Fine-tuning only reduced errors in the PTB dataset but did not improve cross-cohort performance. Integrated Gradient maps confirmed that the model concentrated on QRS onset and T wave offset, supporting physiological plausibility.

**Conclusion:**

*QTcNet*, trained on large-scale algorithmically labelled data, consistently outperformed conventional algorithms across three independent, external validation datasets. Fine-tuning of *QTcNet* may adapt the model to the characteristics of specific cohorts but reduces external validity in other cohorts. We openly release the full model and code, along with a ready-to-use online implementation at https://qtcnet.uni-muenster.de, facilitating further research and community-driven improvement.

## Graphical Abstract

**Graphical Abstract:**
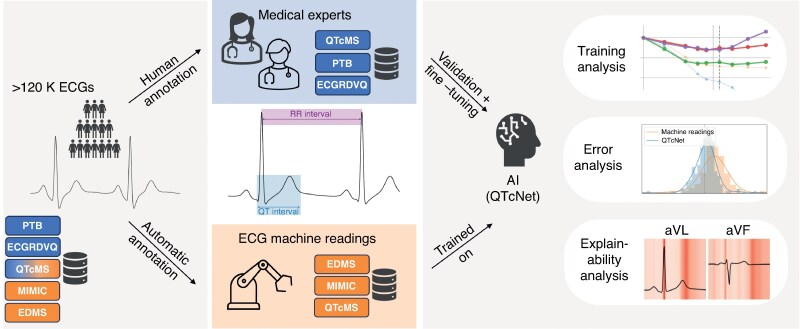

What’s new?

- Automated QT interval measurements are often inaccurate, yet clinicians widely rely on them.
- Our proposed deep learning model for improved QTc interval estimation halved the overall measurement error compared to standard ECG analysis software in three independent external validation cohorts.
- Explainability analyses confirmed model focus on QRS onset and T offset, suggesting physiological plausibility.
- The full model and code are released open source, facilitating future research and community-driven improvement.

## Introduction

The electrocardiogram (ECG) records the electrical activity of the heart and is mainly composed of the P wave, QRS complex, and T wave. The P wave represents atrial activation (depolarization), the QRS complex reflects ventricular depolarization, and the T wave indicates ventricular repolarization. The duration of ventricular de- and repolarization is captured by the interval between the onset of the QRS complex and the end of the T wavethe QT interval (Graphical Abstract).^1^ Accurate measurement and interpretation of the QT interval are crucial because both its shortening and prolongation is associated with life-threatening ventricular arrhythmia.^2^ Ion channel disorders and numerous medications can induce QT prolongation, making manual QT interval measurements a routine task in hospitals around the world. Because QT duration is dependent on heart rate, it is typically corrected using various formulas to normalize the interval to a standard heart rate of ∼60 beats per minute.^3,4^

Computer programs interpreting 12-lead ECGs, including automated QT interval measurements, have been available for more than 50 years.^5^ However, due to imperfect algorithmic annotations, artefacts, or recording errors, results must still be reviewed by an experienced clinician. Despite these well-known inaccuracies and the associated risks of incorrect QT estimations, empirical evidence has shown that clinicians highly rely on these computer annotations.^6^ Although the quality of automated ECG output has been consistently questioned, data in this regard remain outdated and scarce. In the 1970s–1980s, few studies conducted direct comparisons of ECG programs.^7–9^ Willems *et al.*^10^ were the first to directly compare computer with clinician interpretations, using 1220 ECGs and nine different ECG programs. They documented significant deviations, but external validity was limited as they only included ECG records from patients with structural heart disease. More contemporary studies focusing directly on automated ECG interval measurements continue to highlight their unreliable accuracy, indicating that different annotation algorithms often produce discrepant results and that automated QTc measurements tend to overestimate manual assessments performed by experienced cardiologists.^11–13^ Therefore, clinicians should not rely solely on automated QTc measurements, and there is a need to better understand and mitigate the potential systematic biases of current ECG programs in clinical practice.

Recent advances in artificial intelligence (AI) have significantly improved automated ECG evaluation. Numerous studies have used AI methods for detecting various cardiovascular conditions, including QT-related disorders such as long QT syndrome.^14–16^ In contrast, only few studies have directly targeted the more precise estimation of ECG-derived intervals. For example, Diaw *et al.*^17^ investigated several deep learning architectures for automated QT measurements in clinical trials, focusing on single-lead ECG recordings and classification-based methods. Similarly, Tarabanis *et al.*^18^ proposed a convolutional neural network (CNN) that predicts QTc intervals specifically from ECGs with atrial fibrillation by incorporating waveform features, demographic variables, and algorithmically derived measurements from the MUSE algorithm (GE Healthcare).

The present study aims at developing an open-source regression-based CNN model for automated direct QTc estimation using routine clinical 12-lead resting ECGs. By training and validating the model across diverse cohorts annotated with different methods, we seek to overcome existing limitations and to demonstrate the potential of CNN-based regression to improve QTc assessment accuracy and improve clinical applicability.

## Methods

Our study leverages five distinct datasets of 10 s 12-lead resting ECGs to develop, train, and validate the proposed *QTcNet* model. The datasets, which vary in size, annotation methods, and patient populations, provide a diverse foundation for model training and validation (*Table 1*). The datasets, model architecture, training, and evaluation process are described in the following paragraphs. We adhere to the EHRA AI checklist that is provided as a supplementary file.^19^

**Table 1:** Overview of ECG datasets used in this study

	MIMIC	EDMS	PTB	QTcMS	ECGRDVQ
Source	PhysioNet	Internal	PhysioNet	Internal	PhysioNet
Number of ECGs	568 801	63 150	547	210	5219
Gender distribution					
Male	282 363 (49.6%)	35 921 (56.9%)	391 (71.5%)	107 (50.9%)	2637 (50.5%)
Female	282 420 (49.7%)	27 228 (43.1%)	146 (26.7%)	103 (49.1%)	2582 (48.5%)
Unknown	4018 (0.7%)	1 (< 0.1%)	10 (1.8%)		
Mean age ± SD	62.43 ± 16.76	58.37 ± 19.32	56.65 ± 14.20	65.58 ± 17.23	26.97 ± 5.36
Dataset splits	Train: 60 150	Train: 60 150	Fine-tuning: 447	Validation: 210	Validation: 5219
	Test: 3000	Test: 3000	Validation: 100		
Algorithm	Various vendors	MUSE	—	MUSE	—
Expert annotation	—	—	Yes	Yes	Expert oversight

### Datasets

#### MIMIC-IV-ECG

The Medical Information Mart for Intensive Care (MIMIC)-IV-ECG database is a large, publicly available repository containing 800 000 12-lead resting ECG recordings from 160 000 unique patients.^20^ These ECGs were collected from the intensive care unit and emergency department at Beth Israel Deaconess Medical Center in Boston. The dataset encompasses a high variability of clinical conditions and signal characteristics of ECG machines from various manufacturers (including Burdick/Spacelabs, Philips, and General Electric). As model training relied on automatically annotated QTc interval labels, strict filters based on the annotated intervals were implemented to exclude ECGs with presumed lower label accuracy. Specifically, ECGs with automatically annotated intervals outside normal physiological bounds (PR <20 or >500 ms; P wave <20 or >300 ms; QRS <50 or >300 ms; QT <250 or >600 ms) were excluded, resulting in 568 801 ECG recordings with available machine measurements within the pre-specified ranges. Of those, 3000 ECGs were randomly split out for internal testing and monitoring the training process (*Table 1*).

The QRS start, T end, and RR interval were used to calculate QT and QTc intervals with Bazett’s formula:


$$\begin{array}{c} QTc=\;\frac{T_{end}-QRS_{start}}{\sqrt{RR}}. \end{array}$$

This formula for QT interval correction was used because it is the most widely used and the default output of the GE MUSE system.

#### EDMS

The Emergency Department Münster (EDMS) dataset comprises 63 150 12-lead resting ECGs from the emergency department at the University Hospital Münster.^21^ Each ECG was annotated with QT/QTc intervals using the automatic annotation system provided with the GE MUSE software (version 8.0.2.10132), which uses Bazett’s formula by default. In accordance with the MIMIC-IV-ECG dataset, EDMS ECGs whose QTc values fell outside the range of 250–600 ms were excluded, resulting in 60 150 ECGs with available machine measurements within the pre-specified range. Of those, 3000 ECGs were randomly split out for internal testing and monitoring the training process.

#### PTB Diagnostic ECG Database

The PTB Diagnostic ECG Database includes 549 publicly available 12-lead resting ECG recordings, each annotated withQRS onset and T offset by five different cardiology experts that were used for QT interval computation.^22,23^ Because this dataset does not contain expert-annotated R peaks for heart rate correction, the NeuroKit2 algorithm was used for R peak detection.^24^ The median interval of all detected R peaks was used for QTc calculation. For R peak detection, each lead was preprocessed using NeuroKit2 signal cleaning algorithms. Two ECGs of this dataset were excluded due to missing expert annotations and undetectable R peaks.

One hundred randomly selected PTB ECGs were reserved for external validation (PTB validation set). The remaining 447 ECGs (PTB fine-tuning set) were used to test the effect and impact of fine-tuning of the trained *QTcNet* model on expert-annotated labels.

#### QTcMS

The QTcMS dataset, specifically built for this study, comprises 210 12-lead resting ECGs from the University Hospital in Münster, randomly selected from all cases presented between 24 September 2022 and 14 June 2023. This dataset was completely reserved for external validation. The dataset includes the automated QTc annotations from the MUSE system (same as EDMS) but was complemented with manual expert annotations. Specifically, a cardiology expert visually identified the onset of a global QRS complex across all leads, determined the end of the T wave primarily using the tangent method in lead II (or alternative leads if necessary), and marked two consecutive R peaks within the same heartbeat. Based on these manual delineations, the corresponding QTc values were calculated using Bazett’s formula.

The availability of both annotation types (machine and expert) enabled a detailed evaluation of the *QTcNet* model by allowing direct comparisons with machine and expert measurements. Additionally, the dataset included diagnostic information about specific ECG abnormalities [premature ventricular complexes (PVC), presence of atrial fibrillation (AFib) or atrial flutter (AFlu), abnormal T waves (inverted or flat T wave), or complete bundle branch block (BBB)] that were used for subgroup analyses. Consequently, the QTcMS dataset served as the gold standard for assessing the precision and reliability of *QTcNet*.

#### ECGRDVQ database

The ECGRDVQ database comprises semi-automatically annotated 12-lead resting ECG recordings acquired from a prospective randomized controlled clinical trial investigating drug-induced changes in cardiac repolarization (ECG effects of ranolazine, dofetilide, verapamil, and quinidine, NCT01873950).^25^ In this study, 22 subjects received an hERG potassium channel blocker (dofetilide) or one of three multichannel drugs (quinidine, ranolazine, or verapamil), with ECGs recorded at 16 distinct time points. At each time point, three optimal ECGs were extracted using Antares software (AMPS, New York, NY) based on criteria of stable heart rate and maximum signal quality, ensuring accurate capture of early and late repolarization phases. QT intervals were measured semi-automatically with expert oversight. The deliberate selection of high-quality ECGs in the controlled study setting may render this dataset less representative of the variability encountered in routine clinical practice. Therefore, ECGRDVQ was not used for model training but was reserved to provide broader external validation.

#### Ethical approval and informed consent

Collection and analysis of the EDMS and QTcMS datasets was approved by the responsible medical ethics committee (Ärztekammer Westfalen-Lippe, approval no. 2022-494-f-S and 2022-429-f-s) under a waiver of informed consent in accordance with state law for health data privacy (§6 Abs. 2 GDSG NW). The MIMIC-IV-ECG, PTB, and ECGRDVQ datasets are publicly available research resources accessible via PhysioNet.^26^

### Machine learning model and training process

The development of the presented machine learning model was based on a CNN architecture tailored for multivariate time series data. The following sections outline the architecture, training procedures, and evaluation methods used for QTc estimation.

#### Model architecture

In our study, the InceptionTime architecture, a CNN optimized for time series classification tasks, was used due to its demonstrated capabilities to effectively capture essential temporal dependencies and multiscale features in ECG signals, making it particularly suitable for robust QT interval prediction.^27,28^ InceptionTime uses multiple Inception Modules, each processing the input data at varying temporal scales. To adapt the architecture for QTc estimation, the classification head was replaced with a regression-based output layer, enabling the model to predict continuous QTc values instead of discrete classes. The model was implemented with Python, using the PyTorch library for deep learning and the tsai package for model implementation.^29^

#### Training procedure

**Figure 1:**
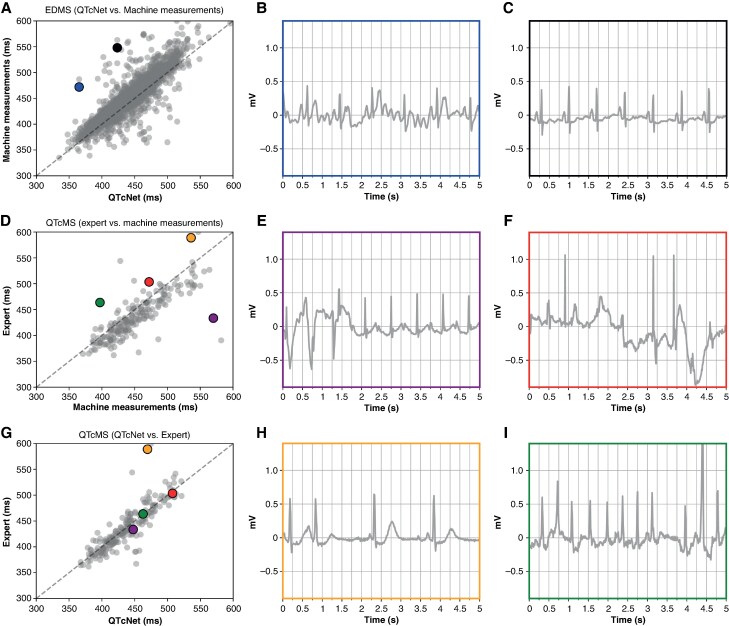
Comparison of algorithm-based QTc estimates (machine measurements), QTcNet predictions, and expert annotations on the QTcMS and EDMS datasets. Panel *A* shows QTcNet predictions (*x*-axis) vs. machine measurements (*y*-axis) on EDMS; panel *D* shows machine measurements (*x*-axis) vs. expert annotations (*y*-axis) on QTcMS; and panel *G* presents QTcNet predictions (*x*-axis) vs. expert annotations (*y*-axis). Each point corresponds to an individual ECG, with the diagonal line marking perfect agreement. Six outlier ECGs are highlighted in blue, black, purple, red, yellow, and green on the scatter plots. Their corresponding waveforms (lead II) are displayed in panels *B*, *C*, *E*, *F*, *H*, and *I*, each with borders in the matching colour. Outliers were reduced with QTcNet despite high levels of noise and artefacts (panel *G*).

**Figure 2:**
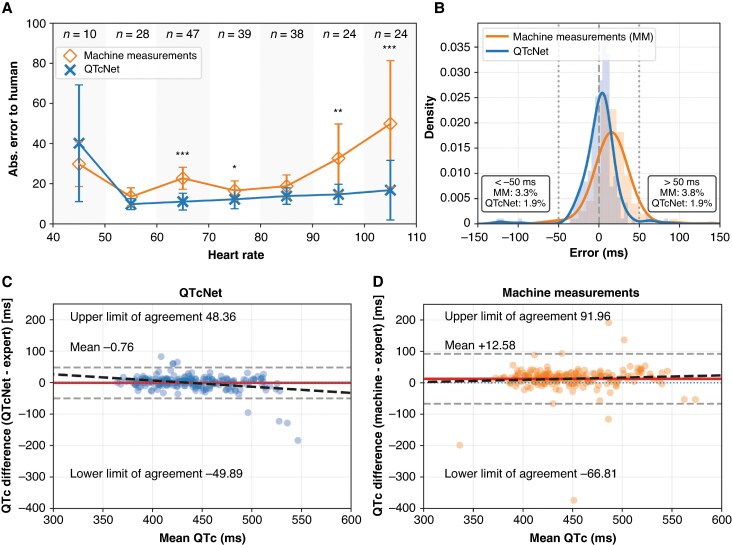
Direct comparison of QTcNet performance compared to machine measurements in QTcMS. Panel *A* shows the mean absolute error of QTcNet predictions and machine measurements vs. human expert annotations across varying heart rates. Error bars indicate 95% confidence intervals. Stars denote the outcome of the appropriate two-sided paired test applied to each interval (paired Student’s *t*-test when the paired differences passed the Anderson–Darling normality test, otherwise Wilcoxon signed-rank): *P < 0.05, **P < 0.01, ***P < 0.001. Panel *B* compares the spread of error of QTcNet (blue) and machine measurements (orange) vs. expert annotations, illustrating both a systematic overestimation and a wider spread of error with machine measurements compared to QTcNet. Bland–Altman plots illustrate pairwise agreement of QTcNet (panel *C*) and machine measurements (panel *D*) vs. expert annotations. Horizontal dashed lines indicate upper and lower limits of agreement.

For training of the *QTcNet* model, two algorithm-annotated datasets (MIMIC and EDMS) were integrated to provide a large, diverse set of ECGs. The input data for model training were the raw 12-lead ECG waveform data represented as a 12 × 1000 matrix (12 leads with a sampling frequency of 100 Hz over a period of 10 s) and algorithm-based QTc measurements as a continuous variable in ms. Due to a systematic overestimation bias in the EDMS algorithm-based QTc measurements (see *Figures 1D* and *2*), a constant of 15 ms was subtracted from all EDMS QTc values. During the training phase on the MIMIC and EDMS datasets, model performance was monitored as a function of the number of training samples. Learning curves were plotted to indicate a saturation point at which the inclusion of additional ECGs yielded minimal improvements. Increasing imbalance between the MIMIC and EDMS datasets negatively impacted overall model performance. Therefore, only a random subset of 60 150 MIMIC-IV-ECGs was included for final model training to provide a 1:1 balanced training set from MIMIC and EDMS. During training, an L1-loss function, the Adam optimizer, and a *ReduceLRONPlateau* strategy for learning rate adjustments (initial 0.001) were used, with training capped at 100 epochs. Early stopping terminated training if validation loss did not improve for ten consecutive epochs. In addition to the dual-source *QTcNet* model, three single-source models were trained on (i) MIMIC data only, (ii) EDMS original data, and (iii) EDMS with overestimation bias correction, to evaluate the benefit of combining different data sources for model training in comparison to single-source approaches.

#### Model fine-tuning

After the initial training process, the *QTcNet* model was fine-tuned using expert-annotated ECGs from the PTB fine-tuning set to elucidate how the model could be adapted to specific cohorts and annotation standards. Fine-tuning proceeded by continuing training from previously computed weights, employing the same training procedure as initial training, but with an initial learning rate of 0.0001.

### Performance evaluation

Single-source models, *QTcNet*, and the fine-tuned model were evaluated using the mean absolute error (MAE) and root mean square error (RMSE) against expert-annotated QTc intervals from the PTB Diagnostic ECG Database (100 validation ECGs), the QTcMS dataset (210 ECGs), and the ECGRDVQ dataset (5219 ECGs). The performance of algorithmic QTc measurements (machine measurements) and direct comparisons between *QTcNet* and machine measurements were quantified only in the QTcMS dataset that included both algorithmic and expert annotations. MAE of machine measurements and *QTcNet* against expert annotations were compared at different heart rates (40–49 bpm, 50–59 bpm, 60–69 bpm, 70–79 bpm, 80–89 bpm, 90–99 bpm, and ≥100 bpm) to evaluate heart rate dependent performance. For each interval, the paired differences (machine or *QTcNet* error to expert error) were first tested for normality with the Anderson–Darling test (*α* = 0.05). If normality was not rejected, a two-sided paired Student’s *t*-test was used to compare differences between groups. Otherwise, a two-sided Wilcoxon signed-rank test was applied. To complement error-based metrics, agreement between *QTcNet* and machine measurements vs. expert annotations was assessed using intraclass correlation coefficients (ICC; two-way consistency model) and Bland–Altman limits of agreement.^30^

### Explainability

To establish intuitive explainability of the *QTcNet* model, we employed Integrated Gradients (IG), a method that quantifies the relevance of input features for neural network outputs.^31^ IG importance maps were computed individually for each full 10 s ECG in the test set to illustrate which segments of the ECG waveform mainly influenced model predictions. For dataset-wide assessment of each ECG segments’ contribution, IG maps were aggregated at the lead level, averaged across the entire test cohort, and aligned with median beats as previously described.^21,32^ Although IG maps do not provide a definitive explanation of individual feature impact, averaging them across the entire test set enables the identification of consistently relevant regions within the ECG waveform.^33^

## Results

Automated QTc measurements by the MUSE system provided by GE (‘machine measurements’) showed a consistent overestimation (≈15 ms) of QTc intervals compared to expert annotations (*see Figure 1D*). The *QTcNet* model reduced this systematic bias, aligned the scatter distribution along the diagonal, and reduced the number of outliers even in the presence of noise and artefacts (*Figure 1E,F,I*). However, there remained outliers where the model annotated values near the mean QTc interval of 450 ms (*Figure 1G*). *Figure 1H,I* shows a representative case of an outlier ECG (yellow dot in *Figure* *1D* and *G*), where the *QTcNet* model produced an unclear QTc estimate.

### Systematic error analysis of machine measurements, single-source CNN models, and *QTcNet*

*Table 2* summarizes a detailed performance comparison between standard machine measurements, single-source CNN models (trained on MIMIC or EDMS), and the proposed *QTcNet* model (trained on the combined MIMIC and EDMS datasets). Performance was measured as the MAE and RMSE across the three independent expert-labeled datasets (PTB, QTcMS, and ECGRDVQ), which provided clinically based gold standard annotations for external validation. Standard machine measurements yielded an MAE of 23.40 and RMSE of 40.13 against expert annotations on the same ECGs. When correcting for the observed systematic bias of −15 ms, both values decreased to MAE = 18.05 and RMSE = 37.79.

**Table 2:** Comparative performance of QTc estimation models, each trained on different data bases, validated across three expert-annotated datasets

Model	Metric	PTB	QTcMS	ECGRDVQ	Average
Machine measurements (original data)	MAE		23.40		23.40
	RMSE		40.13		40.13
Machine measurements (15 ms bias correction)	MAE		18.05		18.05
	RMSE		37.79		37.79
MIMIC model	MAE	21.27	17.89	7.83	15.66
	RMSE	30.43	27.04	**11.00**	22.82
EDMS model (original data)	MAE	29.42	20.68	17.13	22.41
	RMSE	36.91	28.81	18.80	28.17
EDMS model (15 ms bias correction)	MAE	19.04	14.70	7.96	13.90
	RMSE	**28.85**	26.27	12.08	22.40
QTcNet (EDMS + MIMIC)	MAE	**18.84**	**13.88**	**7.42**	**13.38**
	RMSE	29.61	**24.85**	11.78	**22.08**
Fine-tuning (QTcNet -> PTB)	MAE	16.04^a^	14.76	9.02	13.27^a^
	RMSE	27.60^a^	25.84	14.53	22.66

Performance is measured as the mean absolute error (MAE) and root mean squared error (RMSE) against expert annotation (gold standard benchmark). Bold values highlight the best performing model per dataset.

^a^indicates improved performance with fine-tuning on the PTB dataset.

The single-source CNN models generally outperformed the machine measurements and hence their original training data. Both the MIMIC single-source model and the EDMS single-source model (with bias correction before training) yielded similar results with the best performance observed in the ECGRDVQ dataset (see *Table 2*). The single-source model trained on EDMS data without bias correction performed worse across all expert-annotated datasets but still yielded lower error on expert-labelled ECGs compared to the machine measurements (see *Table 2*).

By integrating both the MIMIC and the bias-corrected EDMS dataset into a single model, the proposed *QTcNet* either outperformed or matched the best single-source models. Notably, in terms of MAE, *QTcNet* achieved the best performance on every dataset, with average scores of MAE = 13.38 and RMSE = 22.08.

Model fine-tuning on 445 expert-annotated ECGs from the PTB dataset did not improve cross-cohort model performance (MAE marginally better at 13.27, RMSE marginally worse at 22.66). After fine-tuning, MAE and RMSE were only reduced in the PTB test set but increased in the remaining datasets (*Table 2*).

### Improvement analysis with *QTcNet* over standard machine measurements

Direct comparison of *QTcNet* and machine measurement errors relative to expert annotations in the QTcMS dataset revealed that *QTcNet* approximately halved the machine measurement error. MAE was reduced from 23.4 ms with standard machine measurements to 13.4 ms with *QTcNet* and RMSE was reduced from 40.1 to 22.1 ms. Similarly, ICC showed higher global agreement between *QTcNet* and expert annotations (ICC 0.819) than between machine measurements and expert annotations (ICC 0.621).

*QTcNet* performance was independent of heart rate, while there was a clear trend of greater errors of machine measurements with increasing heart rate (see *Figure 2A*). In absolute terms, conventional machine readings showed a mean signed error of 12.58 ms (systematic overestimation), while *QTcNet* was virtually unbiased at −0.27 ms (*P* < 0.001 by Wilcoxon signed-rank test). Furthermore, the proportion of ECGs with a large error of >50 or less than −50 ms was reduced from 7.1% with machine readings to 3.8% with *QTcNet* (*Figure 2B*). Consistently, Bland–Altman plots illustrated less outliers and generally narrower limits of agreements between *QTcNet* and expert annotations compared to the machine measurements (*Figure 2C,D*).

**Figure 3:**
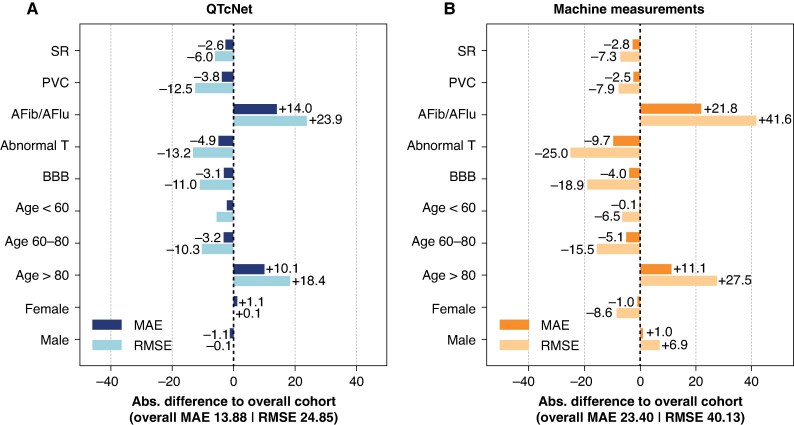
Subgroup performance of QTcNet (panel *A*) and machine measurements (panel *B*) in the QTcMS dataset. Mean absolute error (MAE) and root mean squared error (RMSE) is given for each subgroup as the absolute difference compared to the overall cohort. SR, sinus rhythm; PVC, premature ventricular complexes; AFib/AFlu, atrial fibrillation or atrial flutter; BBB, bundle branch block.

Subgroup analyses confirmed consistent *QTcNet* performance, irrespective of gender or the presence or PVC, complete BBB, or abnormal T waves (*Figure 3A*). However, performance was lower with atrial fibrillation or flutter, and in older patients >80 years with an expected overlap between those two groups (AFib/AFlu prevalence in age <60 years, 8.82%; age 60–80 years, 14.43%; age >80 years, 17.78%). Relative subgroup performance compared to the overall cohort was similar with the machine measurements but with larger baseline errors.

### Learning curve analysis

**Figure 4:**
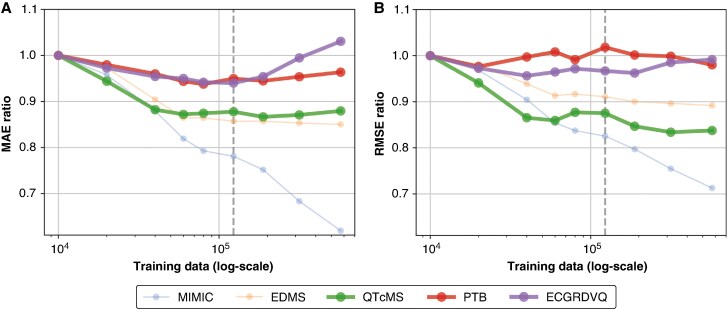
Comparison of model performance as a function of training set size (log-scale). Panel *A* shows the mean absolute error (MAE) ratio and panel *B* shows the root mean square error (RMSE) ratio relative to a start sample size of *n* = 10 000 evaluated on five different sets: MIMIC (blue), EDMS (orange), QTcMS Expert (green), PTB (red), and ECGRDVQ (purple). Expert-based datasets where marked as bold line. The vertical dashed line highlights the last point of balanced EDMS and MIMIC training data.

*Figure 4* illustrates the *QTcNet* performance as a function of sample size, to analyse the dependency of model performance on increasing imbalance between ECGs from MIMIC and EDMS. Each curve corresponds to testing on a different dataset (MIMIC test split, EDMS test split, QTcMS, PTB, and ECGRDVQ), illustrating how model performance changes with increasing sample size and varying proportions of MIMIC vs. EDMS ECGs. The vertical dashed line highlights the last point at which the training set maintained a 1:1 ratio between MIMIC and EDMS samples. As the training data became increasingly dominated by MIMIC, model performance only improved on the MIMIC dataset. MAE and RMSE continuously decreased in MIMIC with increasing sample size, while the model’s MAE on the expert-annotated datasets began to increase and RMSE remained relatively unchanged. Therefore, the final *QTcNet* model was trained on a 1:1 balanced training dataset consisting of 60 150 randomly selected MIMIC-IV-ECGs and all 60 150 EDMS ECGs without subsequent fine-tuning.

### Model explainability

**Figure 5:**
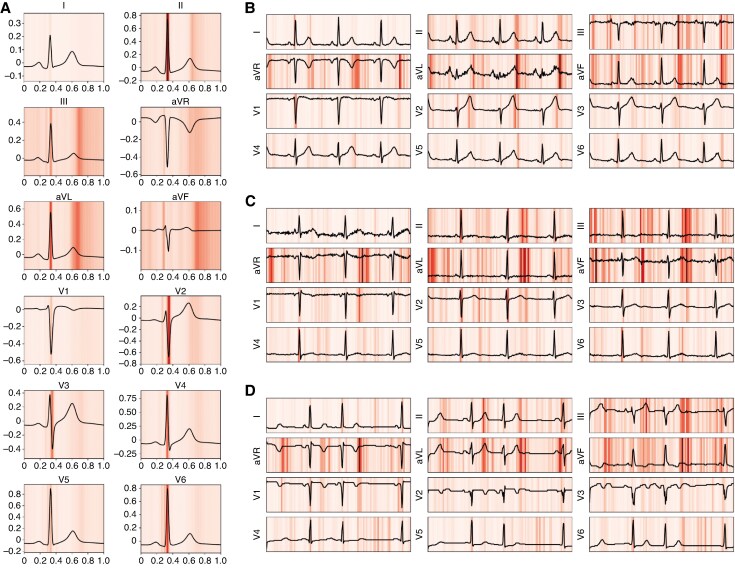
Integrated Gradients (IG) maps illustrating feature relevance for QTc prediction. Panel *A* shows dataset-wide aggregated importance scores computed using IG for each ECG lead and aligned to median beats. Black lines represent the average ECG signals across the test set, while the red overlay highlights regions the model identified as relevant for QTc prediction. Darker areas indicate higher attribution values. Panels *B–D* display three individual test set ECGs alongside their respective importance scores, illustrating how the model’s focus can vary across different signal morphologies.

To establish model explainability at the individual ECG level, *Figure 5B–D* displays three representative test ECGs alongside their corresponding IG importance scores. In all cases, the model tended to attribute relevance to physiologically meaningful waveform segments, with highest attribution values concentrated around the QRS onset, R peak, and T wave offset. This pattern was confirmed in median beats that were averaged across the entire test datasets (*Figure 5A*). Despite variations in signal quality and morphology, the averaged IG maps maintained a clear focus on the R peak, QRS onset, and T offset across all leads, suggesting that QT-relevant features were the primary basis for prediction.

## Discussion

We present a deep learning model capable of direct estimation of heart rate corrected QT intervals from raw input ECG data. The network architecture trained solely on thousands of automatically annotated ECGs consistently outperformed the original annotations it was trained on. Especially the number of extreme outliers was reduced by nearly half from 7.1 to 3.8% (*Figure 2B*). This is clinically especially relevant, as under- or overestimation of the QTc interval by more than 50 ms exposes patients to a clinically unrecognized risk of ventricular arrhythmias (in the case of QTc underestimation) or falsely withholding indicated but potentially QT prolonging medication (in the case of QTc overestimation).

As there is no scientific or clinical consent on which formula might be best for heart rate correction of the QT interval, we directly used QTc values with Bazett’s formula for model training to provide a rough estimate of the ‘true’ QTc interval and let the model discover its own relationship between heart rate and QT. Indeed, our explainability analyses confirmed a consistent model focus on QRS onset and T offset that are relevant for QT calculation and R peaks for heart rate correction. With this information, the model produced accurate QTc estimations across a wide spectrum of heart rates (*Figure 1A*). Therefore, even in the absence of a definitive ground truth and using imperfect QTc labels instead, the network correctly extracted underlying patterns and learned robust representations that generalized well to unseen data. This finding is consistent with recent research suggesting that deep neural networks possess an inherent capacity to filter out noise and leverage imperfect labels when high-quality annotations are hard to obtain.^21,34–37^ In scenarios where manual labelling is expensive or impractical, an extension to noise-robust learning strategies could enable the practical use of large-scale but noisy datasets.^38^

Our findings reproduce earlier reports of significant discrepancies of automated QTc estimation from various vendors when compared to manual expert annotations.^11,13^ In accordance with Alqarawi and Allwaim,^13^ we observed a general overestimation, with a mean signed error of 12.58 ms (MAE of 23.40 ms), and significant variability due to outliers, as evidenced by a high RMSE of 40.13 ms. Neumann *et al.*^11^ found a similar overestimation of automated QTc estimations compared to manual measurements only when using the tangent method but not the threshold method, suggesting that this observed systematic bias might reflect differences in the method of T offset determination.

We demonstrate that elimination of the overestimation bias and balancing data distributions between EDMS and MIMIC ECGs improved the performance of the proposed *QTcNet* model. This model is the closest model to expert-based annotation in our tests in terms of MAE and on par to the single-source models in terms of RMSE. This suggests an advantage of training a model on multiple sources that incorporate different annotation algorithms and patient cohorts when dealing with noisy training labels.

Despite these improvements, some outliers remain, as illustrated in *Figure 1*. When model outputs are unstable, the loss function tends to pull predictions towards the average QTc value to reduce overall loss but leading to inaccurate estimates for individual atypical signals. However, this behaviour may be leveraged to flag potentially unreliable QTc estimations by comparing the CNN output with other automated algorithms. If the model annotates a mean QTc of ≈450 ms with a significant discrepancy to other annotations algorithms, this could create a safeguard to alert clinicians when measurements should be reviewed or repeated.

As evidenced in our subgroup analyses, outliers mostly occurred in ECGs with prevalent atrial fibrillation or flutter, potentially driven by irregular RR intervals, whereas *QTcNet* performance remained stable in the presence of PCV, BBB, or abnormal T wave configurations. The lower error observed in the ECGRDVQ dataset further suggests that the presence of noise or artefacts may influence model performance, as this dataset consists exclusively of high-quality ECGs from a controlled study setting, whereas artefacts regularly occur in the other datasets. However, this observation may be biased by the younger age of the ECGRDVQ participants and the fact that the dataset comprises multiple ECGs per participant resulting in lower overall variability of ECGs within the dataset. Furthermore, *Figure 1D–I* illustrates improved *QTcNet* performance over machine measurements even in the presence of noise or artefacts.

Although initial training of *QTcNet* did not include any expert-annotated data, fine-tuning the system on 445 expert-annotated ECGs from the PTB dataset did not improve average model performance. A performance gain was only observed in the remaining PTB ECGs with the cost of reduced external validity (greater error in QTcMS and ECGRDVQ), suggesting that fine-tuning on a limited number of samples from a single source may result in overfitting without improving overall model performance.

Our approach has several limitations: first, the amount of expert-labelled data was limited. Fine-tuning was performed on only 445 ECGs from a single source, which may have introduced specific biases into the model, resulting in the reduced external validity. More expert-annotated data would be needed to stabilize a CNN-based QTc model. Second, the black-box nature of CNNs implies that we cannot fully explain why the system produces a specific QTc measurement. In this context, rigorous validation, especially with critical edge cases, is essential. As shown in *Figure 5*, an approximate explainability analysis reveals that the model consistently attends to the QT interval region across all leads, yet the precise decision-making process remains non-transparent.

## Conclusion

We developed and externally validated a CNN model for improved automated QTc estimation on diverse algorithmically and expert-annotated datasets. Our AI-driven approach demonstrated superior accuracy and reduced systematic biases compared to traditional algorithmic methods. Given the high reliance of clinicians on automated QTc measurements, our model shows great promise in improving the accurate detection of QTc prolongation and reducing potential misdiagnoses. Future studies are needed for validation in larger expert-annotated datasets and for establishing the definite clinical utility of this approach.

## Supplementary Material

euaf274_Supplementary_Data

## Data Availability

All code for model development is being made publicly available via GitHub (https://github.com/imi-ms/qtcnet/) with peer-reviewed publication. We encourage the community to use our code to replicate and extend the findings presented in this work. This open-source software tool is intended solely for research and informational purposes. It is not a medical device and has not been approved or certified by any regulatory agency. ECG data from MIMIC and PTB are publicly accessible on PhysioNet. Raw data from the EDMS and QTcMS datasets cannot be shared publicly due to patient privacy regulations.
